# Role of nitric oxide and its metabolites as potential markers in lung cancer

**DOI:** 10.4103/1817-1737.65036

**Published:** 2010

**Authors:** Fares Masri

**Affiliations:** *Department of Biochemistry and Microbiology, University of Kalamoon, Deratiah, Syria*

**Keywords:** Carcinogenesis, oxidative stress, lung cancer, nitric oxide, protein modification

## Abstract

Nitric oxide (NO) and reactive oxygen species (ROS) play important physiologic roles as mediators of signaling processes. However, high concentrations of NO and ROS result in damage to cellular and extracellular components. Excessive production of endogenous and/or exogenous ROS and NO is implicated in the pathogenesis of lung cancer. NO and its metabolites interact with ROS to generate potent nitrating agents leading to protein nitration, which is one of the several chemical modifications that occur during oxidative/nitrosative stress. Although there is considerable evidence in support of a role for NO in protein modifications and carcinogenesis, recent data suggest that NO has antagonistic cellular effects, leading to either promotion or inhibition of tumor growth. However, the role of NO in tumor biology is still poorly understood. This review demonstrates the role of NO and its metabolites as potential markers in lung cancer.

In spite of a rapid growth in the field of NO research which has attracted scientists from a variety of disciplines, and the recognition that NO may play key roles in a variety of pathologic processes including cancer, research on NO in cancer remains at its infancy. Because of the recognition that NO is a free radical, significant research has been invested into the role of NO in mutagenesis and thus, indirectly, carcinogenesis. A full understanding of the molecular biology of NO synthesis and its subsequent fate is essential in order to appreciate the roles of NO in carcinogenesis, tumor progression and cancer therapy.

## Nitric Oxide and its Biosynthesis

Nitric oxide (NO) is composed of nitrogen and oxygen atoms that covalently bind to each other to form a diatomic molecule. NO is a water- and lipid-soluble gas containing an unpaired electron, making NO a highly reactive molecule that participates in many chemical reactions. NO has a short half-life (few seconds) and a short range of bioactivity. In general, NO is a relatively stable free radical that readily diffuses from the site of production, crossing cell membranes and interacting with targets without the need for special transporters or receptors.[1,2]

The physiology of NO is complex and its role in inflammation is controversial with both anti-inflammatory and pro-inflammatory effects.[3] The first indication that NO played an important role in physiology was its identification as the endothelium derived relaxing factor (EDRF).[4] In this role, the NO that is continuously released from endothelium enters adjacent vascular smooth muscle, where it promotes vessel relaxation though its activation of soluble guanylate cyclase. Since this study, NO has been discovered to play a role in many biologic functions including cell proliferation and angiogenesis, and has also been implicated in variety of diseases such as cancer and primary arterial hypertension.[5–7]

**Figure 1:**
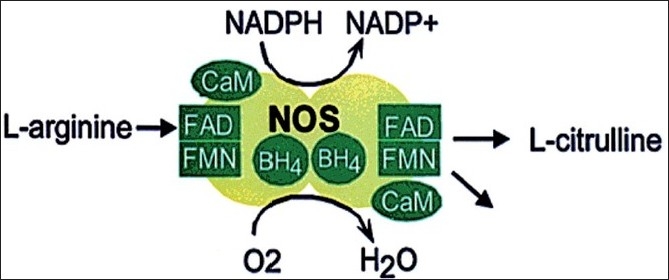
Nitric oxide biosynthesis. NO is synthesized from the conversion of L-arginine into L-citrulline by nitric oxide synthases in the presence of other cofactors including NADPH, FAD, FMN, CaM and BH4

NO is made from L-arginine in a reaction catalyzed by a family of intracellular enzymes called nitric oxide synthases (NOS), in the presence of oxygen and the reduced from of nicotinamide adenine dinucleotide phosphate (NADPH). This reaction requires many other cofactors including flavin adenine dinucleotide (FAD), flavin mononucleotide (FMN), calmodulin (CaM) and tetrahydrobiopterin (BH4)[8] [Figure 1].

## Protein Nitration: Mechanism and Selectivity

Tyrosine residues in protein are susceptible to several chemical modifications including nitration, chlorination and bromination. Tyrosine residues in protein are chlorinated by HOCl to form 3-chlorotyrosine, or brominated by HOBr to form 3-bromotyrosine,[9] or nitrated by NO and its metabolites to form 3-nitrotyrosine (protein nitration).[10,11] Tyrosine nitration can occur when a cell or an organism experiences oxidative stress.[11,12]

**Figure 2:**
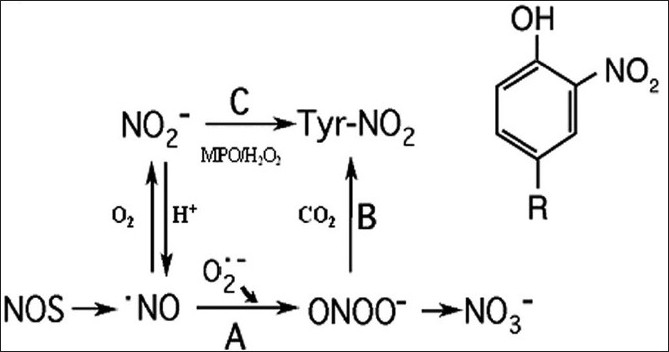
Nitrotyrosine formation in the lung. NO reaction with superoxide generates peroxynitrite (ONOO–) (reaction A). Peroxynitrite and CO_2_ form an adduct that has enhanced nitrating capabilities (reaction B). In the absence of target molecules, NO_3_– forms. NO_2_– formation is relatively slow and may be consumed by peroxidase catalyzed reaction C to generate nitrotyrosine (Tyr-NO_2_) also

A number of pathways have been suggested for the production of 3-nitrotyrosine [Figure 2]. NO undergoes a direct bimolecular reaction with O_2_– yielding peroxynitrite (ONOO–) at almost diffusion-limited rates [Figure 2, reaction A].[13,14] High levels of NO and superoxide favor the formation of peroxynitrite (reaction A). Once formed, ONOO– may nitrate tyrosine (Tyr-NO_2_) by reaction B, or decompose to NO_3_–. NO_2_– may be used to nitrate proteins by a peroxidase-catalyzed reaction (reaction C).[15] However, the data presented to date do not confirm the existence of a single pathway but rather a multitude of biologic reactions could be responsible for the tyrosine nitration.

The process of tyrosine nitration is protein specific and alters select tyrosine residues in the affected protein.[16–20] Several factors could demonstrate this selectivity such as location of nitrating agents from the protein, abundance of the protein, number of tyrosine residues in the protein and position of tyrosine residues in a specific sequence (motif). Nitration of proteins is associated with over 50 diseases including cancer[3,21,22] and linked to many different functional consequences as apoptosis and oxidative stress.

## Role of NO in Carcinogenesis

Despite the important research that has been invested into the role of NO in carcinogenesis, understanding the role of NO from initiation to promotion in a tumor remains incomplete. The effect of NO on tumors appears to depend on two main determinants: the amount of NO generated and the cell type. Excessive amounts of NO increase apoptosis in some tumor cells, whereas lower amounts can increase vascularity and protect the cells from apoptosis.[23] Cytotoxicity of NO varies among different cell types and in different states of differentiation.[24,25]

Although NO has been proposed to be an important mediator of tumor growth,[26] the mechanism by which NO contributes to the promotion of tumor growth is still unclear. The involvement of NO in cancer is complex, leading to either promotion or inhibition of tumor growth.[23] Some studies have shown that presence of NO dramatically reduced aggressiveness of the tumor, whereas others have reported that tumor cell lines became more aggressive.[23,27] Currently, it is believed that high levels of NO may be cytostatic or cytotoxic for tumor cells, whereas low levels may promote tumor growth[27,28]; however, the role of NO in tumor biology is still poorly understood.

## Effect of NO and its Metabolites in Lung Cancer

The effort to reduce lung cancer mortality over the last 30 years has been the most evident and costly failure in the war on cancer. Lung cancer is the most common cause of cancer death. Lung cancer accounts for 28% of cancer deaths and about 6% of all deaths. Lung cancer can be initiated when a normal cell within the lung undergoes genetic mutations, causing it to become an abnormal cell. These abnormal cells can form a tumor that has the potential to invade neighboring blood vessels and it spreads to other places in the lungs.

Tobacco smoke is the main cause for lung cancer and is responsible for 87% of all lung cancers in the United States.[29,30] The risk increases with the amount of tobacco used and the amount of time it has been used. Cigarette smoke, a major source of exogenous oxidants, leads to chronic airway inflammation with accumulation and activation of leukocytes which produce high levels of reactive oxygen species (ROS) and NO. For example, cigarette smoke contains 10^14^ ROS[31–33] and 700 ppm NO per puff.[34,35] Excessive or inappropriate production of endogenous and/or exogenous ROS and NO is implicated in the pathogenesis of lung cancer.[5,6] High levels of oxidative stress have been noticed in patients with advanced lung cancer.[36] Masri *et al*, showed that lung cancer patients have increased exhaled NO compared to healthy controls.[37] Exhaled NO levels were measured by an off-line method according to the established guidelines by American Thoracic Society using a chemiluminescent analyzer (NOA 280, Sievers, Boulder, CO, USA).[38]

Recently, breath analysis, which includes gaseous phase analysis that measures exhaled nitric oxide, and exhaled breath condensate (EBC), has been proposed as a noninvasive and simple technique to investigate neoplastic processes in the airways and for the early detection of lung cancer.[39]

**Figure 3:**
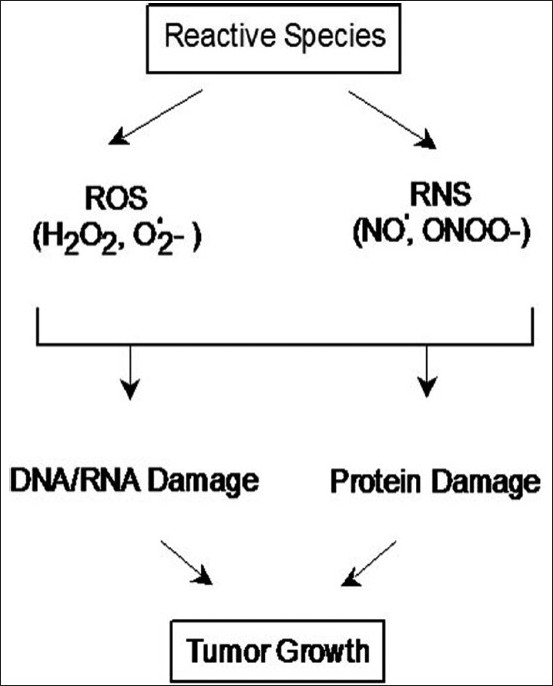
Role of reactive species in tumorigenesis. Multiple exposures of reactive species result in damage to DNA and proteins and thus are potentially carcinogenic in the long term

**Figure 4:**
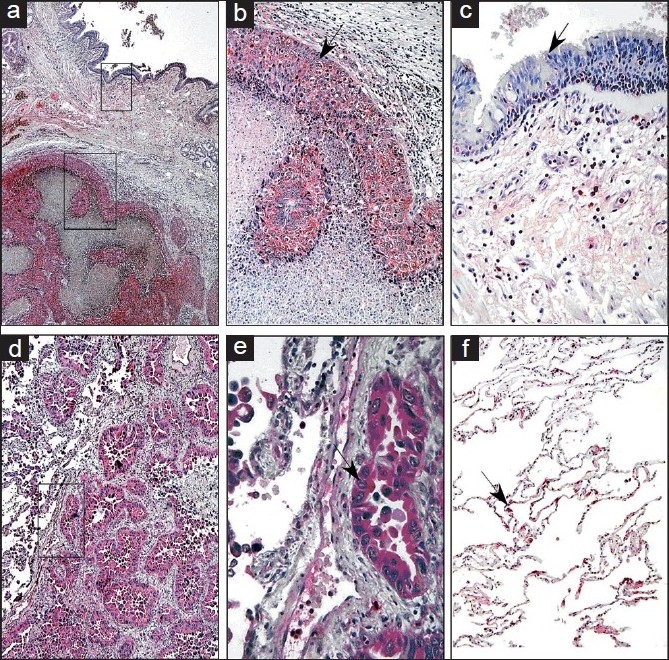
Nitrotyrosine staining in lung cancer. Immunohistochemical staining using an anti-nitrotyrosine antibody demonstrated that the nitrotyrosine-modified proteins were localized mainly to the tumor and not to the surrounding normal tissue. This was observed in squamous cell carcinoma (a, b) as well as in the well-differentiated adenocarcinoma (d, e). Nitrotyrosine staining was mainly observed in the tumor itself (b, e) but not in the adjacent tumor-free lung regions (c) or was weakly reactive in different regions of the lung from the same cancer patients (f) Source: Ref. 37

Nitric oxide by itself is relatively nonreactive.[40] Its damaging properties are derived from its reaction with ROS which may modify protein function through nitration[3,41,42] or cause nucleotide modifications in DNA[42,43] and thus are potentially carcinogenic in the long term [Figure 3]. Lung cancer patients have significantly higher levels of nitrated proteins in serum, supporting the presence of oxidative and nitrosative stress.[44,45] Immunohistochemistry studies have shown that protein nitration is increased in the tumor relative to the tumor-free region in lung cancer patients. The presence of nitrotyrosine appears tightly defined to the boundaries of the tumors [Figure 4].[37] Protein nitration is typically considered to be a marker of oxidative damage rather than as an active agent responsible for pathogenic effects.[16–19] Protein nitration is a specific process affecting select proteins in cells and tissues.[16,17,19,20]

NO and protein nitration may have combined effects on tumorigenesis. NO consumptive mechanisms require oxygen. In the hypoxic environment of the tumor, NO consumption may decrease leading to local increases of NO. Thomas *et al*, have shown that levels of NO determine its biologic effects including angiogenesis, erythropoiesis and glycolysis.[46] At low levels of NO, activation of HIF-1α occurs; but at higher levels, NO inactivates p53, a tumor suppressor that regulates cell cycle genes such as *p*21^waf/cip1^.[47,48] Over 90% of lung tumors are defective in p53, but p53 inactivation by nitration could also contribute to carcinogenesis. Other proteins previously associated with lung cancer such as hnRNPK are also nitrated and they may significantly enhance cell proliferation and anchorage-independent growth.[49] Likewise, nitration of annexin III may affect cell growth and signaling pathways.[50] With broad effects on angiogenesis, glycolysis, p53 activity, antioxidant potential in the lung and alterations in cell growth pathways, NO may create a microenvironment that can initiate tumorigenesis and/or promote tumor heterogeneity leading to metastasis.

## Role of NO and Exhaled Breath in Diagnosing and Monitoring Lung Cancer

Although recent studies show increased level of exhaled nitric oxide (ENO) and its metabolites including protein nitration in lung carcinogenesis, the evaluation of such changes in level of nitration as a potential marker for lung cancer is poorly researched, especially its clinical aspects.

Currently, measurement of ENO has evinced a lot of interest in the diagnosis, treatment and monitoring of asthma. Concentration of exhaled nitric oxide is markedly elevated in asthma, and its elevation is positively related to the degree of eosinophilic airway inflammation and symptoms.[51–53] We believe that ENO and nitrated proteins can also be used as potential clinical markers in lung cancer if further investigations are done.

Some studies listed a number of proteins that were nitrated in cancer including lung cancer.[37,45,46,54] It is known that nitration of some of these proteins, e.g., aldolase and manganese superoxide dismutase (MnSOD), causes loss of their activities.[55] Determination of these target proteins may allow other researchers in the cancer field to evaluate them as potential disease markers and to determine novel pathways that may be involved in carcinogenesis.

Recently, there has been a sudden increase in the number of studies investigating the biomarkers of lung cancer in exhaled breath, using the induced sputum technique which allows sampling of the airways in a noninvasive manner. Published data have shown that exhaled breath contains a pattern of volatile organic compounds (VOCs), which distinguishes patients with and without lung cancer.[56,57] Lately, a simpler method of detecting unique patterns of VOCs has been developed using gaseous chemical sensing devices. Mazzone *et al*, showed by using a colorimetric sensor array that patients with lung cancer present a unique chemical signature of the breath with moderate accuracy.[58] Recent advances in odor-sensing technology have created chemical sensing devices called “electronic noses”. Electronic noses rely on arrays of chemical vapor sensors that respond to specific stereochemical characteristics of an odorant molecule, particularly VOCs.[59] Machado *et al*, showed that the exhaled breath of patients with lung cancer has distinct characteristics that can be identified with an electronic nose.[60] Further, EBC also contains biomarkers of oxidative stress, which is an imbalance between oxidants and antioxidants, associated with lung cancer development.[61]

Although different exhaled biomarkers have been studied in lung cancer patients, further validation studies are required before using them.

It is hoped that this information will encourage and assist government and non-government service providers to do clinical studies in order for exhaled breath analysis including NO measurement to become fully established in the diagnosis and management of lung cancer.

## Conclusion

The prognosis of lung cancer is still poor because of the absence of valid approaches to its early detection. Exhaled breath analysis and ENO measurement may provide useful assays in predicting diagnosis and disease progression.

This review lays the groundwork for future studies to address the role of NO and protein nitration in carcinogenesis. Since the levels of NO are higher in cancer patients and nitration is restricted to the tumor, these suggest novel roles of these mechanisms in the tumor. Further work is required to determine the functional consequences of protein tyrosine nitration and its clinical role in the disease process.
